# Developmental changes in audio-visual speech integration during the first year of life in infants at elevated and typical likelihood of autism

**DOI:** 10.1371/journal.pone.0347046

**Published:** 2026-05-12

**Authors:** Elena Capelli, Maddalena Irma Cassa, Elena Maria Riboldi, Carolina Beretta, Eleonora Siri, Chiara Cantiani, Massimo Molteni, Valentina Riva

**Affiliations:** Scientific Institute, IRCCS E. Medea, Child Psychopathology Unit, Bosisio Parini, Lecco, Italy; Universita degli Studi di Udine, ITALY

## Abstract

Infants’ ability to integrate auditory and visual information, i.e., audiovisual integration (AVI), emerges during the first year and is crucial for effective communication and early development. However, there is limited longitudinal research on how AVI develops across the first year of life, particularly in infants at elevated likelihood of autism (EL). This study aimed to investigate the developmental trajectories of AVI in response to congruent and incongruent speech stimuli in EL and typical likelihood (TL) infants at 6, 9, and 12 months. Using eye-tracking techniques and the McGurk effect paradigm, we explored infants’ preferential looking behavior towards facial features (eyes vs. mouth) and their response to audiovisual congruence. EL infants were then evaluated at 24 months to explore the associations between AVI and later autism-related traits. Across likelihood groups, infants showed a robust developmental shift from greater attention to the eyes at 6 months toward increased attention to the mouth at 9 and 12 months, consistent with expected developmental changes in audiovisual speech processing. Infants also displayed higher mouth preference in the non-fusible mismatch condition at 6 months, suggesting early sensitivity to audiovisual incongruence. Interestingly, EL infants showed a delayed developmental shift toward mouth-looking across the first year of life. Exploratory outcome analyses revealed that infants showing clinical signs of autism at 24 months displayed a flatter developmental trajectory in eyes-to-mouth preference. The present study emphasizes the importance of examining sensory processing trajectories in EL infants, as delayed shifts in attention to the mouth could signal subtle developmental differences that may have long-term implications for subsequent communication skills.

## Introduction

Integrating auditory and visual information in meaningful ways is fundamental for human communication and for navigating the complexities of the environment. From birth, infants are immersed in a multisensory world, where speech, gestures, and facial expressions co-occur. Sensitivity to audiovisual integration (AVI) begins to emerge during the first year of life [1,2] and continues to improve throughout early development as a function of multimodal experience, especially social interaction [3,4]. AVI supports speech perception by allowing infants to associate auditory speech sounds with visual articulatory cues such as lip movements, thereby enhancing their ability to segment speech, detect phonetic contrasts, and eventually learn spoken language [5,6]. This multisensory redundancy is particularly beneficial in noisy environments and is considered a foundation for later language and social development.

A well-established paradigm to study AVI in the speech domain is the McGurk effect [7,8]. In this illusion, auditory and visual syllables are intentionally mismatched to create either a *fusible* percept (e.g., auditory /ba/ with visual /ga/, perceived as “da”) or a *non-fusible* percept (e.g., auditory /ga/ with visual /ba/, perceived as “bga”) [9]. Crucially, evidence indicates that even infants are sensitive to such audiovisual incongruence. Eye-tracking and neurophysiological studies show that by 6–12 months, infants orient differently when confronted with fusible and non-fusible conditions, revealing early emerging forms of AVI [9–11]. Distinct brain responses to fusible versus non-fusible mismatches – particularly in frontal and temporal regions – suggest that infants’ brains already differentiate audiovisual congruence and incongruence during the first year [9].

Importantly, AVI development is intertwined with infants’ shifting attention to specific facial features. Over the first year, infants’ preference for the mouth versus the eyes follows a developmental trajectory often described as an *inverted U-shape*: at 4 months infants predominantly attend to the eyes, between 6–12 months attention shifts increasingly toward the mouth, peaking around 12 months, and subsequently declining as gaze returns to the eyes [2,5,12]. Looking to the mouth has been associated with later expressive vocabulary [13–15], highlighting its developmental relevance. Thus, both AVI and shifting gaze to internal facial features represent crucial processes in early language acquisition.

In autism, audiovisual integration has been extensively studied, revealing differences in how individuals process audiovisual synchrony and incongruence. Autistic individuals often show reduced sensitivity to audiovisual synchrony, weaker susceptibility to the McGurk effect, and atypical patterns of sensory processing [16–19]. Meta-analyses confirm a consistent reduction in the McGurk susceptibility among autistic individuals compared to controls [20,21]. Notably, these differences appear more pronounced in childhood than in adulthood, suggesting developmental changes and possible compensation over time [22,23].

While much research has focused on diagnosed autism, there is growing evidence of early AVI differences in infants at elevated likelihood (EL) for autism, particularly infant siblings of autistic children. Eye-tracking and EEG studies suggest that EL infants may show less differentiation between congruent and incongruent conditions and reduced mouth-looking during audiovisual mismatch [10,11,24,25]. For example, Guiraud et al. (2012) found that at 9 months, typical likelihood (TL) infants oriented more to the mouth during mismatch trials, indicating detection of incongruence, whereas EL infants did not show this differentiation. Similarly, Riva et al. (2022) reported that TL infants at 12 months exhibited stronger ERP responses in the left temporal region during mismatch conditions, whereas EL infants responded similarly across conditions [11]. Together, these findings suggest that early differences in AVI may be observable in infants with a familial likelihood for autism.

These observations align with “sensory-first” frameworks, which propose that early alterations in low-level sensory processing may cascade into differences in higher-order socio-communicative abilities [26]. In line with this view, Piven et al. (2017) emphasize how early sensory and attentional processes can act as developmental entry points for autism, potentially setting the stage for later-emerging social differences [27]. Similarly, Johnson et al. (2021), within the AMEND model, highlight how early-stage perceptual and sensorimotor processes may modulate later neurocognitive systems, with downstream consequences for social and communicative development [28]. During the first year, rapid developmental shifts occur in both sensory processing and attention to facial features, making this period particularly critical for understanding how AVI trajectories unfold [24,29–31]. Yet, despite the importance of this period, there is still limited longitudinal research examining AVI in EL infants, particularly in response to congruent versus incongruent speech cues. Cross-sectional studies provide valuable results [9,11], but only longitudinal studies can capture the dynamic developmental changes across infancy.

A recent longitudinal study by Lozano and colleagues (2024) examined AVI in EL infants using synchronous versus asynchronous talking faces at 4, 8, and 12 months. They found that EL infants showed reduced mouth preference at 12 months and no developmental increase in mouth-looking across the first year. However, this study did not directly test responses to congruent and incongruent syllables, leaving open the question of how AVI develops in contexts where audiovisual cues explicitly mismatch [24].

To address this gap, the present study longitudinally assessed AVI in EL and TL infants at 6, 9, and 12 months using eye-tracking with the McGurk paradigm. We had three aims: 1) to investigate whether processing of congruent and incongruent audiovisual stimuli differs between EL and TL infants across the first year; 2) to examine longitudinal changes in mouth-to-eyes preference across groups; and 3) to explore early audiovisual integration and face-scanning trajectories in relation to 24-month clinical outcomes.

Based on prior research, we expected TL infants to show an overall increase in mouth preference across the second half of the first year of life, together with emerging sensitivity to audiovisual incongruence. Previous studies further suggest that EL infants may show reduced attention to the mouth and weaker differentiation between congruent and incongruent stimuli. However, given the limited longitudinal evidence, we hypothesized that EL infants might exhibit a slower or different developmental trajectory in these attentional shifts over time, rather than a qualitatively different pattern. While exploratory in nature, we hypothesized that infants presenting clinical signs of autism at 24 months might show differences in early face-scanning patterns over development.

## Methods

### Participants

The sample was recruited within an ongoing longitudinal project to monitor developmental trajectories in the first years of life and identify early autistic features in EL infants [32–36].

The current study initially included 38 EL and 63 TL infants recruited at 6, 9, and 12 months. The EL group consisted of infants with at least one sibling with a clinical diagnosis of autism [37]. They were recruited at MEDEA Autism Babylab (Scientific Institute IRCCS Medea) thanks to collaboration with the Italian Network for Early Detection of Autism Spectrum Disorders (NIDA Network); [35,38–40]. The TL group was recruited by local advertisement for inclusion in a larger ongoing longitudinal study [41,42]. Four participants were initially recruited but were later excluded from the analyses because they did not engage in the experimental procedure at any time-points, either due to issues with fussiness or withdrawal from the study.

The final sample consists of 38 EL infants and 59 TL infants. Among them, 42 infants participated at all three time-points, including 22 EL infants (57.8% of the EL group) and 20 TL infants (33.8% of the TL group). In addition, 25 infants participated at two time-points (9 EL infants, 23.7% of the EL group; and 16 TL infants, 27.1% of the TL group), and 30 infants participated at one time-point (7 EL infants, 18.4% of the EL group; and 23 TL infants, 38.9% of the TL group).

Inclusion criteria for both likelihood (LH) groups were: 1) gestational age ≥ 36 weeks; 2) birth weight ≥ 2000 g; 3) absence of known genetic abnormalities or neurological conditions; 4) absence of major complications during pregnancy and/or delivery likely to affect brain development; 5) absence of clinically significant sensory impairments, based on developmental screening; 6) both parents were native Italian speakers; and 7) usable eye-tracking data in at least one time-point (see Eye-tracking data preprocessing and analysis section).

The two groups did not differ for sex distribution (TL: 32 males, 28 females; EL: 17 males, 21 females; χ2(1) =.688; p = .407). Descriptive statistics for age at each time point are shown in Table 1. The present study was conducted according to the Declaration of Helsinki guidelines, with written informed consent obtained from parents for each infant before data collection. The individual in this manuscript has given written informed consent (as outlined in PLOS consent form) to publish these case details. All procedures were approved by the Ethical and Scientific Committee at the Scientific Institute IRCCS Medea, Bosisio Parini, Lecco, Italy. This study was conducted from 2022/09/26–2024/09/26.

**Table 1:** Descriptive statistics for age at each time-point in TL and EL infants.

		TL (n = 59)			EL (n = 38)		
	*N*	*Mean (SD)*	*Range*	*N*	*Mean (SD)*	*Range*	*t-test* *(p-value)*
**Age at T6** (months)	35	6.44 (0.38)	(5.8, 7.5)	25	6.40 (0.41)	(5.8, 7.4)	.338 (.736)
**Age at T9** (months)	39	9.56 (0.45)	(8.7, 10.6)	30	9.51 (0.47)	(8.7, 10.5)	.380 (.705)
**Age at T12** (months)	41	12.48 (0.43)	(11.7, 13.5)	36	12.49 (0.50)	(10.9, 13.6)	−.134 (.894)

### Eye-tracking stimuli

#### Preferential looking McGurk paradigm.

At 6, 9, and 12 months, infants participated in the eye-tracking preferential looking paradigm based on the McGurk effect. Previous research on Italian samples has shown a more pronounced McGurk effect when an auditory bilabial phoneme is paired with a visual non-labial phoneme [43], leading to the selection of the /pa/ and /ka/ combination to induce a robust illusory effect [11]. Thus, two videos of a female speaker articulating the phoneme /pa/ and /ka/ were recorded. Video stimuli (1920x1080 pixels) were digitized at 25 frames per second and converted into  .mp4 files, while the audio was recorded at a sampling rate of 48 kHz (318 kbps). The muted videos were synched and presented side-by-side with only one audio track, so that one face articulated the congruent syllable, while the other face articulated the corresponding incongruent syllable.

**Fig 1:**
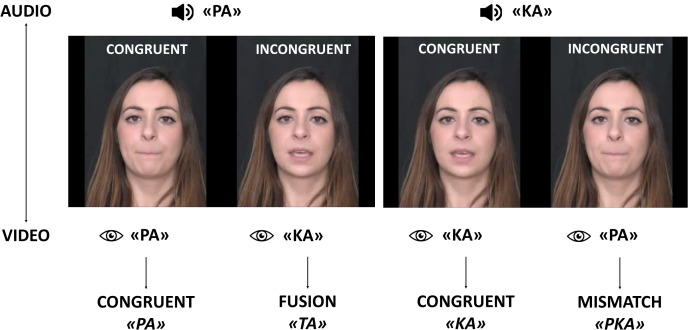
Schematic representation of the McGurk paradigm‌‌.

In the fusion condition (FU), the auditory /pa/ track was selected thus the congruent face (visual PA, auditory /pa/) articulated the /pa/ syllable while the incongruent face (visual KA, auditory /pa/) resulted in the “ta” illusory percept. In the mismatch condition (MM) the auditory /ka/ track was selected thus the congruent face (visual KA, auditory /ka/) articulated the /ka/ syllable and the incongruent face (visual PA, auditory /ka/) produced the non-fusible “pka” percept (Fig 1).

The experimental structure consisted of 8 trials with 10 stimulus repetitions (each stimulus lasting 1800 ms) – 4 trials per condition. The faces position was counterbalanced, ensuring an equal number of congruent and incongruent videos appearing on each side (2 blocks with incongruent faces on the left and 2 on the right for each condition). The order of block presentation was pseudorandomized and kept consistent across infants.

### Eye-tracking procedure

Gaze data were collected using a Tobii ProSpectrum eye-tracker with a sampling rate of 300 Hz. During the task, Tobii ProLab software was used for stimuli presentation and eye movement recording. Infants’ behavior was continuously monitored during the recording sessions using two cameras. During the procedure, infants were seated on their parents’ laps, positioned approximately 60 cm in front of a 24” monitor (1920 x 1820 pixels).

The testing session began with an infant-friendly calibration routine involving two points, strategically placed at corners of the screen (upper left and lower right), and four points were used for validation.

Between each experimental block, infants’ attention was directed towards a central attention-getter (a video of a geometric revolving star accompanied by a ringing sound), which persisted until central fixation was achieved. The next block was manually activated by the experimenter. The stimuli presentation lasted approximately 144 seconds, while the entire procedure took around 4 minutes, as it may include multiple calibration attempts and variable duration of the manually controlled attention-getting routine.

### Eye-tracking data preprocessing and analysis

For each trial, total duration of fixation on all areas of interest (AOIs) was extracted using Tobii Prolab default fixation filter (> 60 msec). Three oval AOIs were drawn for the two faces (see Supplementary materials: Figure S2 in S1 File): eyes region, mouth region, and face region. AOIs were static and sized to include the target region in all frames of the trial for moving features (i.e., mouth).

Further, data processing and trial selection were performed with a custom R script – R version 4.4 [44]. To control for potential effects of gaze quality in the analyses, trials were included in the analyses if both the following conditions were met: a) the infant had inspected both faces, i.e., at least one fixation was recorded on both faces’ area of interest; b) the infant attended the stimuli for a relevant portion of the trial, i.e., fixation time on the faces > 4500 msec (equal to 25% of the overall trial duration). A total of 1672 trials were collected and 1330 were retained (80%) after trial rejection. All trials were excluded for 5 recordings (2 EL and 3 TL). On average, infants contributed 6.46 out of 8 (80.7%) valid trials per session (SD = 1.76; range = 1–8), with comparable retention rates across groups (*t*(204) = − 0.2, *p* = .844; EL: M = 6.48, SD = 1.80; TL: M = 6.43, SD = 1.74). For addi*t*ional information on the trial rejection procedure (number of rejected trials in each time-point and groups; see Supplementary materials: Table S1 in S1 File).

### Clinical outcome at 24 months: Autism diagnostic observation schedule (ADOS-2)

Autistic traits at 24 months were assessed using the Autism Diagnostic Observation Schedule–Second Edition (ADOS-2), a standardized semi-structured assessment of social communication and restricted/repetitive behaviors. Calibrated Severity Scores (CSS; range = 1–10) derived from the ADOS-2 total score were used to characterize symptom severity while accounting for age and language level [45]. For exploratory analyses, the EL sample was subdivided based on the ADOS-2 total CSS at 24 months into infants without clinical signs of autism – EL AUT- (CSS < 4; n = 25) and infants showing clinical signs of autism- EL AUT+ (CSS ≥ 4; n = 11) with this threshold reflecting the range indicative of clinically relevant autism-related traits [45].

### Plan of analyses

Before testing the main hypotheses, we conducted a preliminary analysis to assess potential differences in overall looking time across time-points and likelihood groups. This step was necessary to identify possible confounding effects and to determine whether looking time should be included as a covariate in the subsequent models. A Preliminary General Linear Mixed Model (GLMM) estimated using Restricted Maximum Likelihood (REML) was employed to assess the effects of LH Group (EL, TL), Age and their interaction on looking time at faces. The model included both subject-level (id) and trial-level (trial number 1–8) random effects to account for variability across participants and trials. The use of GLMM allows us to make full use of the longitudinal structure of the data by incorporating all available observations. This approach is well suited for unbalanced designs and missing data and enables the modeling of developmental trajectories over time without imposing restrictive inclusion criteria based on complete participation [46]. The inclusion of Age as a continuous variable allows full trajectories estimation leveraging on the full variability.

In line with our study aims, we first examined whether EL and TL infants differed in their processing of congruent and incongruent audiovisual speech stimuli across time (Aim 1). We then investigated longitudinal changes in eye–mouth preference as a function of LH Group, Age, Condition, and Congruence (Aim 2).

For aim 1, a GLMM estimation (*Model 1*) was calculated for incongruent/congruent preference (i.e., percentage of time spent looking at the incongruent face/incongruent+congruent) for LH Group (EL, TL), Age and Condition (FU, MM), and interaction effects, accounting for individual and trial random effects. For Aim 2, a GLMM (*Model 2*) was conducted to explore eye/mouth preference (i.e., percentage of time spent looking at the eyes/eyes+mouth) testing for LH Group (EL, TL), Age and Condition (FU, MM), Congruence (Congruent, Incongruent), and all possible interaction effects. This model also included random effects at the individual and trial levels. Finally, an exploratory GLMM (*Model 3, Aim 3*) was fitted to investigate eye/mouth preference differences between Outcome groups (TL, EL AUT-, EL AUT+) keeping all other random, fixed and interaction effects as in Model 2.

Models 1, 2, and 3 also included looking time to the faces as a covariate, as differences in looking time at the faces across time-points emerged in the preliminary analyses, ensuring that any observed preferences were not confounded by differences in looking time at the faces.

As the models estimated assume linear Age related trends, additional analyses were conducted including Time-point as a factor (instead of age) to allow for non-linear trends to emerge. All models yielded comparable results (see Supplementary Materials).

Given the complexity of the mixed-effects models, we conducted a conservative power analysis focusing on the primary objective of testing differences between the EL and TL groups. Since data collection followed a mixed longitudinal and cross-sectional design, the sample size was estimated for a single time-point. Using a two-sided two-sample t-test with a significance level of α = 0.05 and a power of 80% (β = 0.20) to detect a large-sized effect (Cohen’s d = 0.7), a sample size of 34 participants per group was estimated (group sample size mean = 34.33; SD = 5.92). The analysis was conducted using G*Power (version 3.1.9.7). Statistical analyses were performed with R version 4.4 and in particular the following packages: lme4 [47] for mixed-model estimation using default identity link function and reporting fixed effects F-tests, performance [48] for R-square estimation and *emmeans* [49] for interaction inspection procedures.

## Results

Firstly, we examined differences in looking time towards the stimuli (i.e., time spent looking at the faces on the screen), the model revealed a significant main effect of Age (F(1, 1318.3) = 5.12, p = .024) with an increase in looking time towards the stimuli with Age (β = 79.9). Neither the main effect of LH Group (F(1, 704.43) = 0.54, p = .463) nor LH Group × Age interaction effect (F(2, 1318.32) = 1.97, p = .161) were statistically significant. The model explained 40.0% of the variance in face looking duration when including random effects (conditional R² = .400), while the fixed effects alone explained only 0.7% of the variance (marginal R² = .007). Despite the small marginal R², the full model provided a significantly better fit than the null model (χ²(3) = 7.96, p = .047).

### Incongruent vs. congruent face preference

For incongruent vs. congruent face preference (i.e., percentage of time spent looking at the incongruent face relative to the total time spent looking at faces), the model did not reveal any significant main or interaction effects. The main effects of LH Group (F(1, 1315.30) = = 0.006, p = .939), Age (F(1, 1317.41) = 1.00, p = .274), and Condition (F(1, 362.80) = 1.69, p = .319) were not statistically significant, nor were any of the interaction effects (Table 2). Participants showed no systematic preference on average (M = 50%), with negligible between-participant variability (singular fit when modeling participant random effects). The model explained minimal variance (marginal R² = 0.007).

**Table 2:** General Linear Mixed Models results.

	Model 1		Model 2	
	Incongruent vs Congruent Face Preference		Eyes vs Mouth Preference	
	F (df)	p	F (df)	p
**LH Group**	0.01 (1, 1315.30)	.939	1.52 (1, 94.36)	.221
**AGE**	1.20 (1, 1317.41)	.274	**192.11** **(1, 2546.76)**	**<.001**
**COND**	1.00 (1, 362.80)	.319	0.49 (1, 5.99)	.508
**CONG**	–		0.22 (1, 2464.45)	.640
**Looking time to the face**	0.14 (1, 687.01)	.708	**56.32** **(1, 2341.28)**	**<.001**
LH Group*AGE	0.01 (1, 1315.40)	.932	**7.05** **(1, 2545.95)**	**.008**
LH Group*COND	0.11 (1, 1315.44)	.746	0.70 (1, 2468.54)	.401
AGE*COND	2.56 (1, 1316.29)	.110	**4.839** **(2, 2460.81)**	**.008**
LH Group*CONG	–		0.19 (1, 2464.05)	.665
AGE*CONG	–		0.33 (1, 2464.45)	.569
COND*CONG	–		0.12 (1, 2464.94)	.731
LH Group*AGE*COND	0.24 (1, 1315.32)	.627	0.35 (1, 2469.11)	.556
LH Group*AGE*CONG	–		2.53 (1, 2464.24)	.112
AGE*COND*CONG	–		0.02 (1, 2464.43)	.901
LH Group*COND*CONG	–		1.6840 (1, 2455.97)	.195
LH Group*AGE*COND*CONG	–		0.78 (1, 2464.15)	.377

*Legend.* AGE=Time-point; COND=condition; CONG=congruence.

### Eyes vs. mouth preference

**Fig 2:**
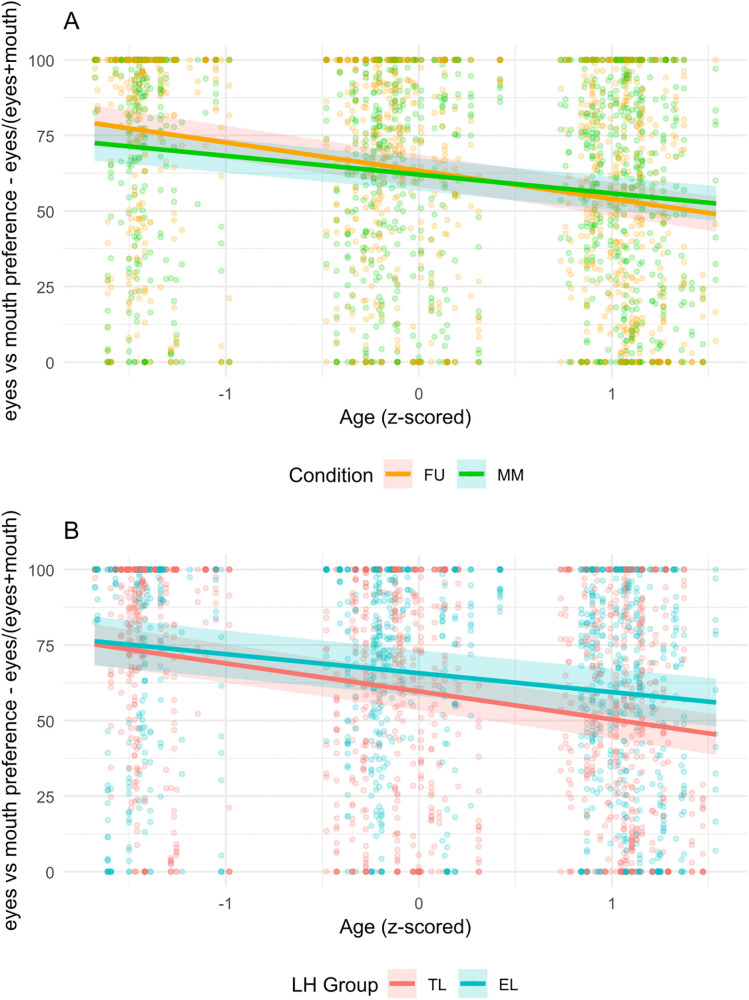
Observed data and estimated marginal slopes for Age*Condition (A) and Age*LH Group (B). Legend. EL = Elevated Likelihood; TL = Typical Likelihood; MM = Mismatch; FU = Fusion.

For eyes vs. mouth preference (i.e., percentage of time spent looking at the eyes relative to the combined time spent looking at the eyes and mouth), a significant main effect of Age was observed, *F*(1, 2464.05) = 192.11, *p* < .001, with a general increase in mouth preference over time (β = − 7.77). The main effect of looking time at the faces was also significant, *F*(1, 2341.28) = 56.32, *p* < .001, suggesting that greater looking time at the faces was associated with stronger mouth preference (β = − 3.93). A significant Age × Condition interaction was observed (Fig 2A), *F*(1, 2470.12) = 9.93, *p* = .002. To probe this significant interaction, simple slopes analyses were conducted (Kenward–Roger degrees of freedom). Results indicated that the Age slopes were significantly negative in both conditions (FU: β = −9.34, *t*(2524) = −12.41, *p* < .001; MM: β = −6.21, *t*(2518) = −8.34, *p* < .001). The slopes differed significantly between conditions, with the FU slope being more negative than the MM slope, Δβ = −3.12).

Finally, a significant LH Group x Age interaction emerged, *F*(1, 2545.95) = 7.05, *p* = .008, (Fig 2B) indicating different patterns of mouth preference shift in the two groups (i.e., EL and TL). Simple slopes analyses indicated that Age slopes were significantly negative across LH groups (TL: β = −9.26, *t*(2560) = −11.71, *p* < .001; EL: β = −6.29, *t*(2509) = −7.90, *p* < .001. The slopes differed between groups with TL presenting a steeper shift towards the mouth compared to the EL group (Δβ = −2.97). The model explained a substantial portion of the variance: Conditional R² = 0.503, reflecting both fixed and random effects, and Marginal R² = 0.075, reflecting fixed effects only. Model comparison indicated that the full model provided a significantly better fit than the null (random only) model, χ²(16) = 263.31, p < .001.

### Exploratory associations between AVI abilities during the first year of life and autism-related traits at 24 months

Model 3 results confirmed the previous model, with significant main effects of looking time at the faces F(1, 2333.27) = 56.85, p < .001 and Age F(1, 2513.61) = 123.26, p < .001, and a significant Condition × Age interaction F(1, 2461.49) = 10.58, p = .001.

**Fig 3:**
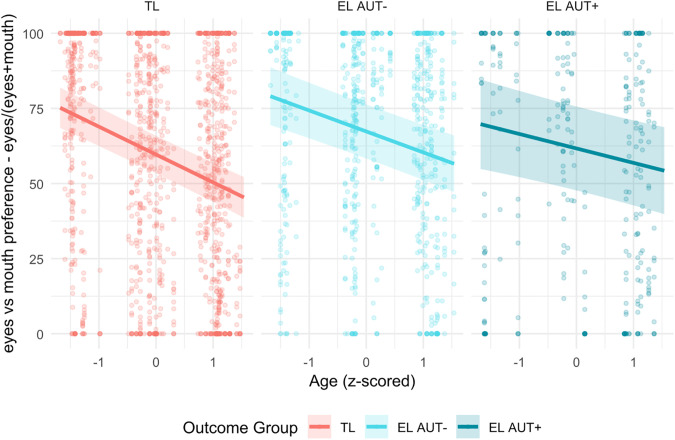
Observed data and estimated marginal slopes for Outcome Group*Age. Legend. EL AUT+ = Elevated Likelihood with clinical signs of autism at 24 months; EL AUT- = Elevated Likelihood without clinical signs of autism at 24 months; TL = Typical Likelihood.

The Outcome Group × Age interaction was also significant, F(2, 2521.38) = 4.26, p = .014. Simple slopes analyses revealed that Age predicted increasing mouth preference across all outcome groups (see Fig 3). The TL slope was steepest (β = −9.26, SE = 0.79, t(2552) = −11.71, p < .001), followed by the EL AUT− group (β = −6.98, SE = 0.96, t(2507) = −7.29, p < .001), and was least steep in the EL AUT+ group (β = −4.81, SE = 1.43, t(2492) = −3.35, p = .001). Comparison of slopes indicated that the EL AUT+ group differed significantly from the TL group (Δβ = −4.45, SE = 1.64, t(2516) = −2.72, p = .018), whereas other pairwise differences were not statistically significant (see Supplementary Materials).

No other main effects or interactions reached significance. The model explained a substantial portion of the variance: Conditional R² = 0.506, reflecting both fixed and random effects, and Marginal R² = 0.079, reflecting fixed effects only. Model comparison indicated that the full model provided a significantly better fit than the null (random only) model, χ²(24) = 271.36, p < .001.

## Discussion

This study aimed to explore differences in developmental trajectories for face scanning patterns between infants at elevated- and typical-likelihood for autism, with a particular focus on how audiovisual processing of congruent and incongruent speech might differentiate the two likelihood groups. Additionally, we aimed to explore, whether EL and TL infants showed distinct patterns in their preferential looking to facial features (such as the mouth-to-eyes preference) in response to the McGurk effect across the second half of the first year of life.

### Incongruent vs. congruent face preference

No significant differences in preference for congruent or incongruent faces emerged between likelihood groups or across time-points. These findings align with results from a previous study using a similar paradigm at 9 months [10], where no differences were observed. This result may suggest that additional specifications, such as preference for specific eyes and mouth regions, may be necessary to detect differences in congruent/incongruent face scanning patterns. Conversely, these findings may contrast with a study examining preferential looking to synchronous versus asynchronous speech, which reported differences emerging around 12 months of age for both groups [24]. A possible explanation for this inconsistency between findings is that asynchrony may be more salient and behaviorally relevant because it disrupts the expected temporal integration of sensory cues. Incongruence alone may not have the same level of impact in this developmental stage. However, it is important to note that further studies are needed to better understand these differences. Notably, Lozano and colleagues (2024) used different stimuli, with an actress uttering a story, while in our case, the incongruence involved mismatched syllables and the McGurk effect. The nature of the stimuli (stories versus syllables) may also play a role in how infants direct their attention toward facial cues.

### Eyes vs. mouth preference

The results of the current study highlight age effect in eyes/mouth preference, with preference moving from the eyes to the mouth from 6 to 12 months. This finding aligns with previous research showing that early on, infants show a particular interest towards the eyes region [5]. The tendency to focus on the eyes during the first months of life serves as a beneficial developmental function, as it plays a critical role in social and communication development.

Conversely, other evidence suggests a shift in visual attention toward the mouth in the second half of the first year, particularly in response to dynamic faces and spoken language [50,51]. Research suggests that while younger infants are more focused on the eyes, by 8–12 months, the mouth becomes increasingly salient, particularly when speech is present or when novel features are introduced, such as non-native language sounds [3,50,51]. This shift is likely connected to the role the mouth plays in language acquisition: infants appear to become more attuned to the mouth area as they begin to process speech sounds and associate them with lip movements, which is crucial for language learning [5,6,25,52,53].

The current study results add to this literature a role of congruency in this developmental shift between eyes and mouth preference. The present study results also revealed a significant time-point by condition interaction, indicating an earlier shift to the mouth in conditions that included non-fusible mismatched (MM) stimuli.

Other studies have supported the idea that processing of audio-visual incongruences emerges early in the first year of life [10,11,54]. Specifically, at 9 months, infants show an increased focus on the mouth when exposed to incongruent audiovisual information [53]. Our findings suggest that the presence of mismatch incongruences may trigger this earlier shift toward the mouth region. It is possible that, in the MM condition, where the perception of one of the stimuli is unfamiliar, the shift in attention differs compared to the fusion (FU) condition. In the FU condition, the incongruent syllable formed by the fusion of visual and auditory cues remains recognizable and meaningful. In contrast, the MM condition leads to a percept that is non-fusible and phonotactically illegal. As a result, this unfamiliarity could lead to a different shift in attention, potentially anticipating the typical progression from focusing from the eyes to the mouth.

This may be particularly relevant considering that, as previously discussed, infants typically show increased attention to the eyes at this stage, but also considering that between 6 and 9 months, a developmental milestone such as babbling normally emerges [55–57]. Conversely, as 12-month-old infants tend to be typically more interested in the speaking mouth, this pattern seems to be true regardless of the condition.

Notably, our results highlight comparable shifts toward both mouths in the MM condition, irrespective of congruence/incongruence, compared to previous research where only the mouth articulating non-fusible percept was more attended [10]. This difference may be attributed to differences in the stimuli presentation. In the current study, a slower stimuli rate presentation (approximately 1 vs 2 stimuli/second) with longer inter-stimulus time may have allowed the infants more time to explore both mouths with reduced competition due to time constraints. Future studies should further investigate mouth preferences across different conditions to assess the influence of other contextual factors (e.g., articulation duration, speech complexity, noise levels).

A further important finding of the present study is that EL infants appeared to show a later trajectory in shifting attention toward the speaker’s mouth. These results offer an alternative perspective compared to previous research, which suggested that EL infants have a lower sensitivity to audiovisual mismatch, pointing to potential atypicalities in integrating auditory and visual information [53]. Previous studies have reported that infants with typical likelihood tend to look longer at the mouth in non-fusible mismatched conditions, particularly at 9 months, whereas this pattern was less evident in EL infants [58]. In contrast, our sample suggests that differences related to family history may emerge more in the overall developmental pathway of eyes/mouth preference, rather than in sensitivity to specific stimuli based on multisensory integration or mismatch. This is particularly interesting given that variations in attentional patterns to the mouth have been linked to different pathways in later socio-communicative abilities [59]. Thus, different timing in the shift toward mouth-looking in EL infants may reflect subtle distinctions in multisensory integration that could influence later development. Since the mouth is the key region supporting audiovisual integration during speech processing, variability in the timing of attention shifts to this region may be relevant for understanding how infants integrate auditory and visual cues during communication.

While EL infants in this study did not show overt differences in their sensitivity to audiovisual mismatch, their overall looking patterns may point to a more heterogeneous developmental trajectory in how they prioritize and process social stimuli.

This interpretation is consistent with previous research on neural responses to audiovisual mismatch in infants, which has highlighted that differences in audiovisual speech processing may be linked more to the maturation of looking behavior than to chronological age [60]. Differences may also appear at different levels of analysis: for instance, neurophysiological responses to mismatch have been observed even in the absence of overt behavioral differences at the same age [9]. Future studies should therefore integrate multiple levels of investigation (e.g., behavioral, neurophysiological) to clarify the mechanisms underlying early audiovisual processing.

Furthermore, evidence of sensory processing differences in the EL population has been reported previously, with some studies showing decreased neurophysiological responses to sensory stimuli [20], while others have documented increased responses [61,62]. These divergent findings suggest that sensory processing in EL infants does not follow a uniform pattern but rather encompasses a range of characteristics. Some studies have proposed that both hypo- and hyper-responsiveness can co-exist within the same child, and that sensory processing patterns may change over the course of development [32,33,63–65]. This variability underscores the complexity of sensory processing in this population, indicating that individual differences likely play a key role in shaping broader developmental trajectories. Sensory heterogeneity may, in turn, influence attentional dynamics and the way EL infants respond to multisensory stimuli. Given that sensory differences are not limited to one type of response, future research should examine how variations in sensory responsivity, whether hypo- or hyper-responsiveness, impact early attentional shifts, particularly in relation to audiovisual stimuli. This may help identify specific integration patterns that are more strongly linked to later socio-communicative outcomes.

It is also important to recognize that infants at elevated likelihood for autism represent a highly heterogeneous population. Differences in sampling characteristics and recurrence rates across cohorts may contribute to variability in observed developmental patterns [57,58]. Moreover, accumulating evidence suggests that multiple developmental pathways and endophenotypes may underlie later autism outcomes [59,60].

In light of this heterogeneity, we conducted exploratory analyses examining developmental trajectories as a function of 24-month clinical outcome.

Exploratory analyses examining outcome groups provided a more fine-grained view of developmental trajectories and suggested that differences in attentional change may be more closely related to infants showing clinical signs at follow-up. Infants who later showed clinical signs of autism (EL AUT+) displayed a flatter developmental slope in the shift from eyes to mouth compared to TL infants, with EL AUT− infants showing an intermediate pattern. Importantly, all outcome groups demonstrated a significant age-related increase in mouth looking, indicating that the overall developmental direction was preserved, while the rate of change differed across groups. Rather than reflecting a qualitatively atypical pattern, these findings may point to differences in the timing or pace of developmental change and provide additional information beyond likelihood group status alone. Within a dimensional framework, the trajectory toward mouth-looking may reflect a slower tuning of attention toward audiovisual speech cues during the first year of life, consistent with sensory-first and developmental cascade models [26,27]. At the same time, given the exploratory nature of this analysis and the relatively small number of infants showing clinical signs at follow-up, these findings should be interpreted cautiously and considered preliminary.

Long-term follow-up studies that track socio-communicative outcomes in EL infants will be critical for disentangling this developmental variability. By monitoring trajectories over time, such studies may clarify whether differences in the rate of early attentional change, rather than differences in developmental direction, are associated with later outcomes, helping to further understand the heterogeneity within the EL population.

Despite the valuable insights provided by this study, several limitations should be considered. First limitation concerns the relatively small number of infants showing clinical signs of autism at follow-up (n = 11). Although this proportion aligns with recurrence rates typically reported in sibling cohorts [57], the small sample warrants cautious interpretation. Nonetheless, these exploratory observations may offer preliminary insights into how early face-scanning patterns and audiovisual processing could vary in infants with emerging clinical signs, providing a starting point for future longitudinal investigations. Second, as is common in infant eye-tracking research, data collection in the first year of life presents inherent challenges, including variable attention, higher movement artifacts, lower calibration accuracy, and increased data loss, which may affect data quality and generalizability. Third, the AV stimuli used in this study may not fully reflect the naturalistic settings in which infants typically interact. Real-world social interactions involve more complex and dynamic stimuli, such as variations in speech, emotional expressions, and contextual cues. Further studies should aim to incorporate more naturalistic stimuli to better capture how infants engage with real-world social information. Finally, although the use of mixed-effects modelling is designed to accommodate for unbalanced samples with missing observations, future longitudinal research should aim to retain a larger cohort with complete participation across all three time-points, thereby strengthening statistical precision and the interpretability of developmental trajectories.

## Conclusion

The main novelty of this work is that it longitudinally addresses developmental pathways of audiovisual processing in response to incongruent and congruent stimuli across three developmental periods during the first year of life in a sample of infant siblings of children with autism.This study provides valuable insights into the developmental trajectories of face scanning patterns and audiovisual processing in infants at elevated likelihood for autism compared to typical likelihood infants. The findings suggest that differences may emerge more in the timing and rate of developmental change rather than in qualitatively distinct patterns, with exploratory analyses indicating flatter developmental trajectories toward mouth-looking in infants showing later clinical signs. These results highlight the importance of considering sensory processing dynamics and developmental timing when studying early variability related to autism. The pattern observed in this study may reflect underlying differences in multisensory integration, which could have long-term implications for language development and social engagement. Early identification of sensory processing differences can inform tailored interventions, offering the potential to improve developmental outcomes and support infants at elevated likelihood for autism.

## Supporting information

S1 File Supplementary Materials 1. Data Quality and Trial rejection description.
(DOCX)

S2 File Supplementary Materials 2. Model 3 statistics table and additional analyses for non-linear trends.
(DOCX)
