# Supplementary material for: Developmental changes in audio-visual speech integration during the first year of life in infants at elevated and typical likelihood of autism
Source: PLoS One. 2026 May 12;21(5):e0347046. doi: 10.1371/journal.pone.0347046 (PMC13166931; doi:10.1371/journal.pone.0347046)

**Supplementary Materials 1 - Developmental changes in the first year of life of audio-visual speech integration in infants at elevated likelihood of autism**

**Data Quality and Trial rejection description**

Calibration metrics indicated good data quality, with a mean accuracy of 0.07° (4.73 pixels) and a mean precision (SD) of 0.30° (15.00 pixels).

**Table S1 –** Rejected & Retained trials for each FH group and Timepoint

| **Rejected – Retained Trial Number (Rejected %)** | | | |
| --- | --- | --- | --- |
|  | **T06** | **T09** | **T012** |
| **TL** | 84 – 204 (32%) | 50 – 264 (16%) | 54 – 272 (16%) |
| **EL** | 68 – 144 (29%) | 50 – 198 (20%) | 36 – 248 (13%) |

**Figure S1 –** Total durations of fixation on the faces histogram (M = 9127.31; SD = 4342.21) – Red line = 4500 ms cut-off (25% of trial ~ < -1 SD from mean).


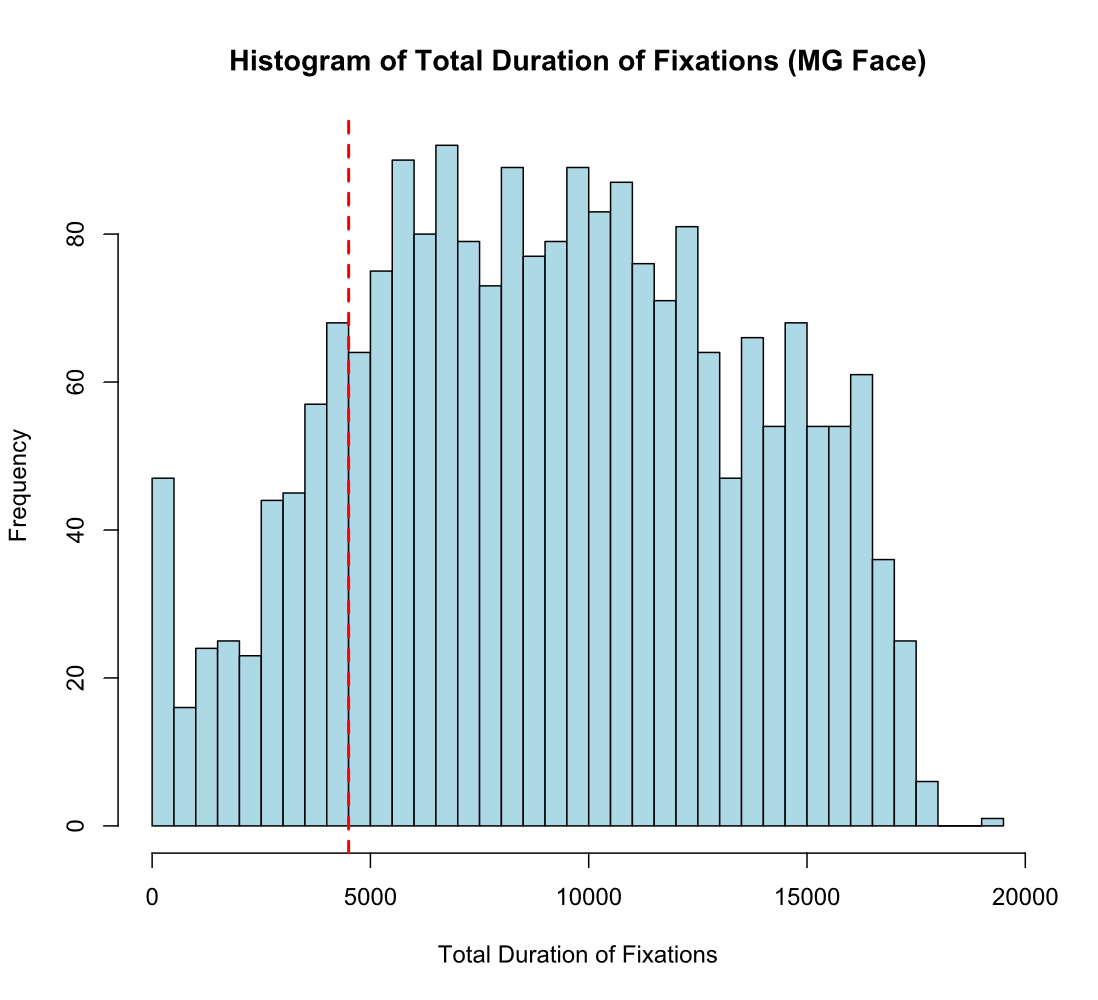

Supplement: S1 File — Data Quality and Trial rejection description. (DOCX) [file pone.0347046.s001.docx]
