# Supplementary material for: Developmental changes in audio-visual speech integration during the first year of life in infants at elevated and typical likelihood of autism
Source: PLoS One. 2026 May 12;21(5):e0347046. doi: 10.1371/journal.pone.0347046 (PMC13166931; doi:10.1371/journal.pone.0347046)
Supplement: S2 File — Model 3 statistics table and additional analyses for non-linear trends. (DOCX) [file pone.0347046.s002.docx]

**Supplementary Materials 2 - Developmental changes in the first year of life of audio-visual speech integration in infants at elevated likelihood of autism**

**Model 3 statistics**

| **Table S1. General Linear Mixed Models results Model 3** | | |
| --- | --- | --- |
| **Effect** | **F (NumDF, DenDF)** | **p** |
| **Face Looking Time** | **56.85 (1, 2333.27)** | **< .001** |
| OUTCOME GROUP | 0.98 (2, 92.42) | .378 |
| CONG | 0.11 (1, 2457.03) | .740 |
| COND | 1.04 (1, 7.58) | .339 |
| **AGE** | **123.26 (1, 2513.61)** | **< .001** |
| OUTCOME GROUP*CONG | 0.22 (2, 2456.84) | .801 |
| OUTCOME GROUP*COND | 0.71 (2, 2460.72) | .494 |
| CONG*COND | 0.34 (1, 2457.40) | .559 |
| **OUTCOME GROUP*AGE** | **4.26 (2, 2521.38)** | **.014*** |
| CONG*AGE | 0.14 (1, 2457.25) | .708 |
| **COND*AGE** | **10.58 (1, 2461.49)** | **.001**** |
| OUTCOME GROUP*CONG*COND | 2.26 (2, 2456.84) | .104 |
| OUTCOME GROUP*CONG*AGE | 1.81 (2, 2456.95) | .164 |
| OUTCOME GROUP*COND*AGE | 0.70 (2, 2461.70) | .499 |
| CONG*COND*AGE | 0.20 (1, 2457.34) | .658 |
| OUTCOME GROUP*CONG*COND*AGE | 0.68 (2, 2456.93) | .506 |

**Additional Analyses investigating non-linear trends – Age modeled as factor (T06; T09; T12)**

Firstly, we examined differences in looking time towards the stimuli (i.e., time spent looking at the faces on the screen), the model revealed a significant main effect of Time-point, F(2, 1302.2) = 3.23, p = .040. Neither the main effect of Group (F(1, 89.1) = 0.61, p = .438) nor Group × Time-point interaction effect (F(2, 1302.1) = 2.23, p = .108) were statistically significant. Post-hoc paired comparisons for Time-point indicated a significant increase in looking time to the stimuli from 6 to 9 months, t(1315) = - 2.19, p = .029, and from 6 to 12 months, t(1317) = - 2.38, p = .017. Conversely, the difference between 9 and 12 months was not statistically significant, t(1287) = - 0.17, p = .867

Model 1 Post Hoc Comparisons – Time-point main effect

| Time-point | Time-point | Difference | SE | t | df | p |
| --- | --- | --- | --- | --- | --- | --- |
| T06 | T09 | -0.0358 | 0.00868 | -4.12 | 1316 | <.001 |
| T06 | T12 | -0.0481 | 0.00850 | -5.66 | 1313 | <.001 |
| T09 | T12 | -0.0123 | 0.00748 | -1.65 | 1302 | 0.099 |

**Incongruent vs. Congruent Face Preference**

For incongruent vs. congruent face preference (i.e., percentage of time spent looking at the incongruent face relative to the total time spent looking at faces), the model did not reveal any significant main or interaction effects. The main effects of Group (F(1, 1312.16) = <0.001, p = .984), Time-point (F(2, 1312.65) = 0.75, p = .472), and Condition (F(1, 6.08) = 1.69, p = .240) were not statistically significant, nor were any of the interaction effects (Table 2).

| **Table S2. General Linear Mixed Models results** | | | | |
| --- | --- | --- | --- | --- |
|  | **Model 1** | | **Model 2** | |
|  | F (df) | p | F (df) | p |
| **Group** | < 0.001  (1, 1312.16) | .984 | 1.2651  (1, 94.46) | .264 |
| **TP** | 0.752  (2, 1312.65) | .472 | **96.716**  **(2, 2523.98)** | **< .001** |
| **COND** | 1.691  (1, 6.08) | .240 | 0.865  (1, 6.15) | .387 |
| **CONG** | - |  | 0.118  (1, 2456.40) | .732 |
| **Looking time to the face** | 0.177  (1, 676.31) | .674 | **57.036**  **(1, 2324.35)** | **< .001** |
| **Group*TP** | 0.347  (2, 1311.38) | .707 | **2.910**  **(2, 2522.57)** | **.055** |
| **Group*COND** | 0.204  (1, 1312.78) | .652 | 0.8243  (1, 2461.04) | .364 |
| **TP*COND** | 1.648  (2, 1312.14) | .193 | **4.839**  **(2, 2460.81)** | **.008** |
| **Group*CONG** | - |  | 0.3896  (1, 2455.96) | .533 |
| **TP*CONG** | - |  | 0.844  (2, 2456.07) | .430 |
| **COND*CONG** | - |  | 0.0721  (1, 2456.77) | .788 |
| **Group*TP*COND** | 0.420  (2, 1311.34) | .657 | 0.088  (2, 2459.67) | .916 |
| **Group*TP*CONG** | - |  | 1.2867  (2, 2456.05) | .276 |
| **TP*COND*CONG** | - |  | 0.117  (2, 2456.24) | .889 |
| **Group*COND*CONG** | - |  | 1.6840  (1, 2455.97) | .195 |
| **Group*TP*COND*CONG** | - |  | 0.3061  (2, 2456.10) | .736 |

*Legend.* TP=Time-point; COND=condition; CONG=congruence.

**Eyes vs. Mouth Preference**

For eyes vs. mouth preference (i.e., percentage of time spent looking at the eyes relative to the combined time spent looking at the eyes and mouth), a significant main effect of Time-point was observed, *F*(2, 2523.98) = 96.717, *p* < .001, with a general increase in mouth preference over time. The main effect of looking time at the faces was also significant, *F*(1, 2324.35) = 57.036, *p* < .001, suggesting that greater looking time at the faces was associated with stronger mouth preference. A significant Time-point × Condition interaction was observed (Figure 2A), *F*(2, 2460.81) = 4.839, *p* = .008. Post-hoc paired comparisons revealed that all differences between time-points across and within conditions were statistically significant (see Supplementary materials: Table S3), except the difference between mouth preference at 6 months in the MM condition and 9 months mouth preference in the FU condition (*t*(21) = -1.22, *p* = .234). In other words, at 6 months’ infants were already more focused towards the mouth region when exposed to a non-fusible condition (MM) with a pattern similar to 9 months olds exposed to a fusible one (FU). This can be further specified by a qualitatively higher preference for the mouth at 6 months in the MM compared to the FU condition that approached statistical significance (*t*(21.2) = 1.91, *p* = .070).

Finally, a marginally significant Group x Time-point interaction was found, *F*(2, 2522.57) = 2.910, *p* = .055, (Figure 2B) indicating potentially different patterns of mouth preference shift in the two groups (i.e., EL and TL). Although the Group x Time-point interaction did not reach statistical significance, we decided to explore it further to investigate potential effects, as previous studies have suggested the importance of examining marginal interactions [48,49]. Post-hoc paired comparisons supported the observed interaction effect: although both groups showed significant increases in mouth preference from 6 to 9 and from 9 to 12 months of age (see Supplementary Materials, Table S4), their developmental trajectories showed some temporal shift. Specifically, mouth preference in the EL group did not significantly differ from that of the TL group at the preceding time point (9-month EL vs. 6-month TL: t(116) = 0.498, p = .620; 12-month EL vs. 9-month TL: t(108) = 0.812, p = .418), suggesting a delayed emergence of the effect in the EL group. The model explained approximately 50% of the variance – R-squared (conditional) = .508.

**Model 3 Post Hoc Comparisons – Time-point ✻ Condition Interaction**

| Time-point | COND | Time-point | COND | Difference | SE | t | df | p |
| --- | --- | --- | --- | --- | --- | --- | --- | --- |
| T06 | FU | T06 | MM | 0.0476 | 0.0249 | 1.91 | 21.2 | 0.070 |
| T06 | FU | T09 | FU | 0.0780 | 0.0194 | 4.02 | 2514.6 | <.001 |
| T06 | FU | T09 | MM | 0.1061 | 0.0247 | 4.30 | 20.3 | <.001 |
| T06 | FU | T12 | FU | 0.2224 | 0.0190 | 11.68 | 2518.3 | <.001 |
| T06 | FU | T12 | MM | 0.1977 | 0.0242 | 8.17 | 18.9 | <.001 |
| T06 | MM | T09 | MM | 0.0585 | 0.0196 | 2.98 | 2508.3 | 0.003 |
| T06 | MM | T12 | MM | 0.1501 | 0.0190 | 7.89 | 2512.2 | <.001 |
| T09 | FU | T06 | MM | -0.0304 | 0.0249 | -1.22 | 21.0 | 0.234 |
| T09 | FU | T09 | MM | 0.0281 | 0.0227 | 1.24 | 14.7 | 0.235 |
| T09 | FU | T12 | FU | 0.1444 | 0.0169 | 8.53 | 2485.5 | <.001 |
| T09 | FU | T12 | MM | 0.1197 | 0.0225 | 5.31 | 14.3 | <.001 |
| T09 | MM | T12 | MM | 0.0916 | 0.0166 | 5.51 | 2482.7 | <.001 |
| T12 | FU | T06 | MM | -0.1748 | 0.0245 | -7.13 | 19.9 | <.001 |
| T12 | FU | T09 | MM | -0.1163 | 0.0227 | -5.12 | 14.7 | <.001 |
| T12 | FU | T12 | MM | -0.0247 | 0.0218 | -1.13 | 12.4 | 0.278 |

*Note.* COND – Condition; MM – Mismatch “pka” condition; FU – Fusion “ta” condition

Model 3 Post Hoc Comparisons – Time-point ✻ Group Interaction

| Time-point | Group | Time-point | Group | Difference | SE | t | df | p |
| --- | --- | --- | --- | --- | --- | --- | --- | --- |
| T06 | TL | T06 | EL | -0.0156 | 0.0533 | -0.292 | 123 | 0.770 |
| T06 | TL | T09 | TL | 0.0948 | 0.0202 | 4.691 | 2551 | <.001 |
| T06 | TL | T09 | EL | 0.0261 | 0.0525 | 0.498 | 116 | 0.620 |
| T06 | TL | T12 | TL | 0.2202 | 0.0200 | 11.022 | 2551 | <.001 |
| T06 | TL | T12 | EL | 0.1367 | 0.0520 | 2.629 | 112 | 0.010 |
| T06 | EL | T09 | EL | 0.0417 | 0.0210 | 1.982 | 2494 | 0.048 |
| T06 | EL | T12 | EL | 0.1523 | 0.0204 | 7.462 | 2501 | <.001 |
| T09 | TL | T06 | EL | -0.1104 | 0.0528 | -2.091 | 119 | 0.039 |
| T09 | TL | T09 | EL | -0.0687 | 0.0520 | -1.322 | 112 | 0.189 |
| T09 | TL | T12 | TL | 0.1254 | 0.0165 | 7.592 | 2513 | <.001 |
| T09 | TL | T12 | EL | 0.0418 | 0.0515 | 0.812 | 108 | 0.418 |
| T09 | EL | T12 | EL | 0.1106 | 0.0180 | 6.132 | 2494 | <.001 |
| T12 | TL | T06 | EL | -0.2358 | 0.0527 | -4.474 | 118 | <.001 |
| T12 | TL | T09 | EL | -0.1941 | 0.0519 | -3.742 | 111 | <.001 |
| T12 | TL | T12 | EL | -0.0836 | 0.0514 | -1.625 | 107 | 0.107 |

*Note.* Family History Group; TL – Typical Likelihood; EL – Elevated Likelihood
